# Development and validation of a novel sodium-overload related genes signature for prognostic prediction in breast cancer: integrating bioinformatics and experimental approaches

**DOI:** 10.3389/fimmu.2025.1653903

**Published:** 2025-10-01

**Authors:** Qizhen Feng, Wenlin Yang, Guohao Su, Fei Wu, Chungen Xing

**Affiliations:** ^1^ Department of General Surgery, The Second Affiliated Hospital of Soochow University, Suzhou, Jiangsu, China; ^2^ School of Clinical Medicine, Jining Medical University, Jining, Shandong, China; ^3^ Department of Pathology, Nantong Tumor Hospital Affiliated to Nantong University, Nantong, Jiangsu, China; ^4^ Institute of Neurobiology, Jining Medical University, Jining, Shandong, China

**Keywords:** breast cancer, sodium overload, NR1H3, prognostic model, therapeutic target

## Abstract

**Introduction:**

Necrosis induced by sodium overload has recently been identified as a novel form of regulated cell death. However, the specific genes associated with sodium overload in breast cancer (BC) remain uncharacterized.

**Methods:**

We identified 753 differentially expressed sodium-overload-related genes (DESORGs) in BC. We performed pathway enrichment analyses, then used univariate Cox regression to select 67 prognostic DESORGs. To build prognostic models, we tested 101 combinations of ten machine learning algorithms. SHAP analysis was used to determine feature importance. Mendelian randomization (MR) was applied to assess causal effects. Experimental validation (in vitro) included overexpression and knockdown studies. GSEA/GSVA and molecular docking were conducted to explore downstream pathways and potential drug candidates.

**Results:**

The ridge regression model showed optimal prognostic power. IFNG was identified as the key feature. The computed risk score was an independent prognostic factor, outperforming traditional clinical variables (AUC = 0.845), and a nomogram built with it yielded good calibration (C-index = 0.815). MR suggested a protective causal effect of NR1H3 in BC, and patients with high NR1H3 expression had significantly better overall survival (p = 0.02). *In vitro*, NR1H3 overexpression suppressed proliferation, colony formation, migration, and invasion, whereas its knockdown had opposite effects. GSEA and GSVA showed that high NR1H3 expression is enriched in immune activation–related pathways. Molecular docking identified Cephaeline and Emetine as potential drugs that upregulate NR1H3 expression.

**Conclusions:**

These findings highlight NR1H3 as a novel DESORG and a promising therapeutic target in breast cancer.

## 1 Introduction

Breast cancer ranks among the top causes of mortality and new cases globally, making it a serious global health concern. With 2.3 million incident cases (11.6% of Pan-cancers) in 2022, breast cancer was the most prevalent cancer diagnosed worldwide. It was also the main cause of cancer-related deaths among women, accounting for an estimated 0.66 million deaths (1). According to current forecasts, there will be 0.87 million BC deaths, and 2.7 million new cases diagnosed globally each year by 2030 (2). Breast cancer mortality is still a problem even with improvements in treatment plans. Patients with distant metastases of breast cancer only had a 5-year overall survival (OS) rate of around 25% (3). Early detection of breast cancer markedly improves the likelihood of successful treatment and patient survival. Thus, identifying new biomarkers for early diagnosis and improved clinical management is urgently needed.

Growing evidence highlights diverse regulated cell death pathways as pivotal players in oncogenesis, including apoptosis (4), ferroptosis (5), cuproptosis (6), and pyroptosis (7). Recently, a unique kind form of necrotic cell death has been discovered, termed necrosis by sodium overload (NECSO) (8). Unlike ferroptosis or cuproptosis, NECSO is uniquely characterized as a form of regulated necrosis initiated by an extreme ionic imbalance—specifically, a massive influx of sodium through channels like TRPM4 that leads to osmotic swelling and subsequent cell rupture. T Koike et al. discovered in 2000 (9) that rat superior cervical ganglion (SCG) cells undergo necrosis and apoptosis *in vitro* when exposed to sodium excess via voltage-dependent Na+ channels. Na+ excess and consequent involvement of the Na+/H+ exchanger cause veratridine neurotoxicity, leading to cytoplasmic acidification and ultimately cell death.

Sodium overload contributes to severe human diseases such as fetal development (10), renal function (10, 11) and cardiac arrhythmias (12). Beyond these conditions, excessive sodium intake can also provoke inflammatory responses. Sodium overload resulted in elevated production of pro-inflammatory markers, including RANTES, NF-κB, Ang II, as well as TGF-β1 (13). Moreover, sodium overload can lead to cell swelling and dilution of intracellular potassium, which are proposed mechanisms for activating the NLRP3 inflammasome which act as crucial element in the process of innate immune response (14). Despite these insights, studies investigating the involvement of sodium overload-associated genes in cancer remain limited. More importantly, high metabolic and proliferative rates of breast cancer cells may render them more sensitive to disruptions in ion homeostasis (15), making the study of sodium-overload pathways a particularly relevant and timely avenue for investigation.

This study aims to uncover promising DESORG candidates for diagnosis and therapeutic intervention in breast cancer by integrating comprehensive bioinformatics analyses and experimental validation. Specifically, this research focuses on the identification of differentially expressed SORGs, the construction of a robust prognostic model using machine learning techniques, and the in-depth investigation of the lead candidate gene, NR1H3 (Nuclear Receptor Subfamily 1 Group H Member 3), to elucidate its functional role and possible application as a treatment target in breast cancer. The discovery of new molecular targets, such as NR1H3, could open new avenues for developing novel therapeutic approaches in breast cancer care.

## 2 Materials and methods

### 2.1 Data sources

The Cancer Genome Atlas (TCGA) website (https://portal.gdc.cancer.gov/) provided the clinical phenotypes and TCGA-BRCA transcriptome data, which included 113 normal samples and 1118 tumor samples. For validation, the GSE199633 dataset, containing microarray data from 637 primary BRAC samples, was obtained from the GEO database (https://www.ncbi.nlm.nih.gov/geo/). This validation dataset was annotated using the GPL15048 platform.

### 2.2 Screening of sodium overload-related genes with differential expression

To identify genes with altered expression related to sodium overload, we searched the GeneCards platform (https://www.genecards.org/) (16) for “sodium overload”. This query retrieved a comprehensive set of 2052 genes, including not only those directly involved in sodium transport (e.g., ion channels, exchangers, and pumps) but also a wide array of downstream effectors and genes implicated in the physiological consequences of altered sodium homeostasis. After processing the gene expression data, we performed differential gene expression analysis. Genes exhibiting an absolute log2 fold change (Log_2_FC) > 0.585 and a *p* < 0.05 were defined as differentially expressed. Heatmaps and volcano plots were generated using the ‘pheatmap’ and ‘ggplot2’ R packages, respectively.

### 2.3 KEGG and GO pathway enrichment analysis

To elucidate the biological activities of all identified DESORGs, “clusterProfiler” R package was performed for KEGG and GO enrichment analyses. Additionally, GSEA was conducted on GO and KEGG gene sets to reveal overall functional enrichment patterns across different experimental groups (17). Hallmark pathway scores were computed per sample using the ‘GSVA’ R package (18).

### 2.4 Development of DESORG-based prognostic models using machine learning

Initial screening via univariate Cox (uni_Cox) regression identified prognostic DESORGs. Subsequently, 101 different modeling approaches were explored by combining ten machine learning algorithms with 10-fold cross-validation using the TCGA-BRCA for our training set. The selected model’s predictive accuracy was independently validated using GSE199633. The model exhibiting the greatest average C-index was selected as an ideal one for further investigation.

### 2.5 SHAP analysis

SHAP assigns an important value to each gene for every prediction by calculating its average contribution across all possible combinations of genes in the model (19). SHAP values were calculated using the Kernelshap R package to quantify each gene’s impact on the model’s output. The resulting SHAP values were illustrated utilizing shapviz R program to enhance comprehension of each gene’s influence on the predicted outcome.

### 2.6 Prognostic analysis of DESORG risk model

Time-dependent ROC analysis assessed the DESORG-based risk model’s prognostic performance, with AUC values quantifying predictive accuracy. Kaplan–Meier survival curves were generated, and differences between groups were evaluated using log-rank tests. Multivariate Cox (mul_Cox) regression analysis was performed to determine independent predictors of prognosis. A nomogram was established to display how the risk score and clinical variables jointly predict survival outcomes. Finally, calibration curves were plotted.

### 2.7 MR analysis

MR analysis was performed using five different MR methods with “TwoSampleMR” R package. Single nucleotide polymorphisms (SNPs) used in this analysis were stringently selected based on the following criteria: strong association with the exposure factor (p < 5e-08), absence of linkage disequilibrium (r² threshold below 0.001 across a 10,000 kb genomic region.), and an F-statistic greater than 10. The IVW method served as the primary approach for inferring causality.

### 2.8 Cell culture and transfection

Human BC cell lines MCF7 and MDA-MB-231 were maintained at 37 °C in an atmosphere containing 5% CO_2_. Cells were cultured in DMEM (Gibco, cat. #11965084) enriched with 10% FBS (Gibco, cat. #10091155) and antibiotic solution (Gibco, cat. #15140163). Small interfering RNAs (siRNAs) targeting human NR1H3, along with a scrambled control siRNA (siRNA-NC), were manufactured by Shenggong Co., Ltd. (Shanghai, China). The siRNA sequences for anti-human NR1H3 were siNR1H3#1: 5’-GCAUCCAGAUAUCUACAAA-3’; siNR1H3#2: 5’-CCACUUCAUGCUGUUGGAA-3’; siNR1H3#3: 5’- GGAAUGCAGCUUCAAGAUG-3’. MCF7 cells were transiently transfected with siRNAs with Lipofectamine RNAiMAX (ThermoFisher Scientific, cat. # 13778030). MDA-MB-231 cells underwent transfection with pENTR221-NR1H3 plasmid (Addgene, cat. # 79514) using Lipofectamine 2000 (ThermoFisher Scientific, cat. # 11668027).

### 2.9 qRT-PCR

Total RNA was extracted from MCF7 and MDA-MB-231 cells using an RNA miniprep kit (Zymo Research, cat. # R1054) and reverse-transcribed into cDNA with PrimeScript 1st strand cDNA Synthesis Kit (Takara, cat. # 6110A). The reverse transcription reaction was carried out at 37 °C for 15 min, then 85 °C for 5 sec to inactivate the enzyme. Subsequently, qRT-PCR was performed with SYBR Green PCR master mix (ThermoFisher Scientific, cat. # A46012) and qPCR was performed with these primers: NR1H3: F 5’-AATGCTGGGGAACGAGC-3’, R 5’-CGGCATTTGCGAAGCCGAC-3’ and β-ACTIN (control): F 5’-ACCATTGGCAATGAGCGGT-3’, R 5’-GGTCTTTGCGGATGTCCAC-3’. Reactions were set up in triplicate for each biological sample. Amplification was carried out on a real-time PCR instrument under the following cycling conditions: Initial denaturation: 95 °C for 2 min; 40 cycles of: 95 °C for 15 sec, 60 °C for 30 sec (annealing/extension); Followed by a melting (dissociation) curve from 65 °C to 95 °C. Gene expression was quantified via 2^-ΔΔCT^ analysis.

### 2.10 Immunoblotting

Total proteins were extracted from cells using RIPA buffer, separated by SDS-PAGE, transferred to PVDF membranes, and blocked with 5% skimmed milk for 1 hour, then subsequently incubated at 4 °C in 5% mike containing primary antibodies NR1H3 (1:5000, proteintech, cat. # 14351-1-AP), and GAPDH (1:10000, proteintech, cat. # 60004-1-Ig) overnight. The membranes were then washed with PBS and incubated with the appropriate peroxidase-conjugated secondary antibodies (1:10000). The images were performed using Immobilon Classico Western HRP substrate (MilliporeSigma, cat. # WBLUC0500) and analyzed with ImageJ software and GraphPad Prism 6. Protein expression levels were standardized to GAPDH expression levels. All Western blot experiments were performed in three independent biological replicates.

### 2.11 CCK8 assay

Cells (5×10³/well) were plated in 96-well plates and incubated for one week. Cell proliferation was evaluated using CCK8 (MeilunBio, cat. # MA0218) at 37 °C for 2 hours to detect absorbance at 450 nm (OD450) with a microplate reader at different time points.

### 2.12 Colony formation assay

A total of 1,000 cells were seeded into each well of a 6-well plate and incubated under standard conditions until distinct colonies became visible. The colonies were washed with PBS, fixed in methanol, and stained with 0.1% crystal violet for 20 minutes. Rinse wells gently with water to remove excess stain and allow plates to air-dry. ImageJ software (v1.53) was employed for automated counting. To ensure accuracy, a size threshold of 50 μm in diameter was established to exclude cellular debris from analysis.

### 2.13 Scratch assay

Cells (1×10^5^/well) were plated in 12-well plates and cultured for 24 h to form monolayers. A sterile 200 µL pipette tip was used to create uniform scratches, followed by PBS washing to remove debris. Fresh medium was then added, and wound closure was monitored by imaging at 0 h and 24 h post-scratching. The migration rate was quantified by measuring the remaining wound area at both time points.

### 2.14 Transwell assay

For migration assays, use uncoated 8 μm pore Transwell inserts (Corning, cat. # 3422). For invasion assays, add 40–50 μL of diluted Matrigel to the upper chamber of each insert, and incubate at 37 °C for 60 minutes to solidify. For both migration and invasion assays, 600 μL of complete medium supplemented 10% FBS was added to the lower chamber, while 200 μL of a cell suspension containing 5 × 10^4^ cells were seeded into the upper chambers of each insert. After 24 hours, remove non-migrated cells from the upper side of the membrane using a cotton swab and fix migrated or invaded cells with 70% ethanol for 10 minutes. Cells were stained with 0.1% crystal violet and then visualized under a phase contrast microscope at 200× magnification in multiple fields to obtain an average.

### 2.15 Drug prediction and molecular docking

Potential drugs targeting NR1H3 were identified using the DSigDB database (https://dsigdb.tanlab.org/DSigDBv1.0/). Drug molecule structures were acquired from PubChem (https://pubchem.ncbi.nlm.nih.gov/), while NR1H3’s 3D structure came from the PDB (https://www.rcsb.org/). Subsequently, protein-ligand blind docking was carried out via the CB-Dock2 platform (https://cadd.labshare.cn/cb-dock2/index.php). This process utilized the CurPocket algorithm, which detects surface curvature-based cavities to predict potential binding sites on the NR1H3 protein, followed by performing blind docking of the selected drug molecules to these identified regions.

### 2.16 Statistical analysis

Data were performed with GraphPad Prism 6 (GraphPad Software) and R 4.2.2 (R Foundation). Data are presented as mean ± SD. For *in vitro* experiments, two-group comparisons used unpaired t-tests; multi-group comparisons employed ANOVA. Three independent biological replicates were performed, and *p* < 0.05 was considered statistically significant.

## 3 Results

### 3.1 Identification of DESORGs and pathway enrichment analyses

**Figure 1:**
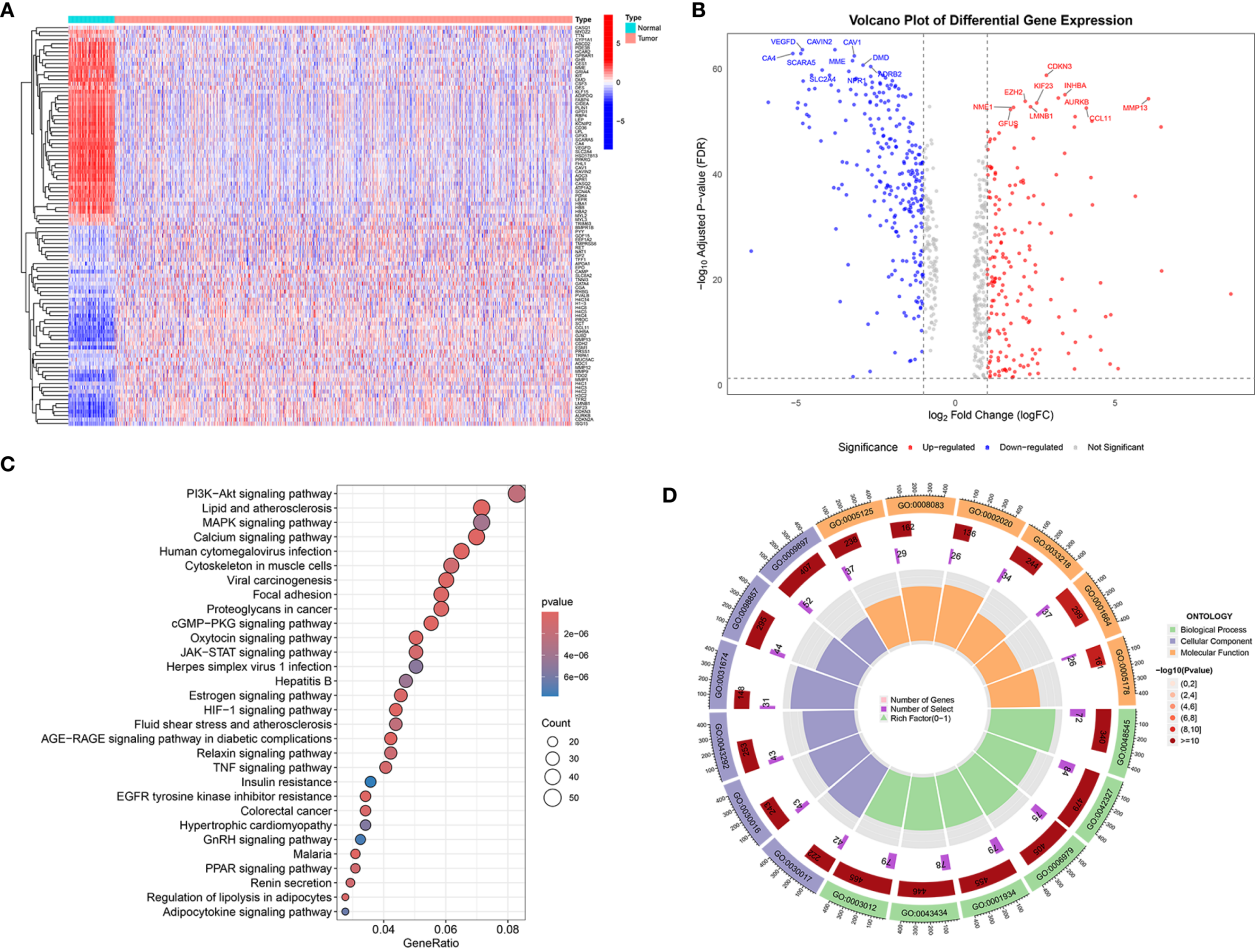
Transcriptomic analysis and functional enrichment of DESORGs. **(A)** Heatmap of DESORGs between normal and tumor samples. **(B)** Volcano plot of DESORGs. **(C)** Bubble plot of KEGG analysis for DESORGs. **(D)** Circos plot of GO analysis for DESORGs.

2052 genes related to the sodium overload pathway were retrieved from the GeneCards database. Differential expression analysis revealed a total of 753 genes exhibited significantly differential expression in tumor tissues relative to norma samples. Among the differentially expressed SORGs, 370 genes showed down-regulated and 383 exhibited up-regulated in tumor samples. A heatmap of DESORGs was shown in **Figure 1A**. The volcano plot in **Figure 1B** highlights both top 10 of down-regulated and up-regulated DESORGs with the most significant false discovery rate (FDR) values. Among the downregulated genes, the most significantly changed were VEGFD, CAVIN2, SCARA5, CA4, CAV1, MME, DMD, ADRB2, SLC2A4, and NPR1. Conversely, the top upregulated genes included CDKN3, INHBA, AURKB, MMP13, EZH2, KIF23, LMNB1, NME1, CCL11, and GFUS. To elucidate the functional implications of all DESORGs, KEGG was conducted and revealed significant enrichment of DESORGs in pathways including PI3K-Akt signaling, calcium signaling, lipid and atherosclerosis, as well as MAPK signaling (**Figure 1C**). Further, GO analysis (**Figure 1D**) indicated that the DESORGs participate in biological processes such as the positive regulation of phosphorylation, response to steroid hormone, and response to oxidative stress. In terms of cellular components, DESORGs were involved in the sarcomere, myofibril, and contractile muscle fiber. Regarding molecular function, the DESORGs showed enrichment in cytokine activity, growth factor activity, and protease binding.

### 3.2 Construction and evaluation of machine learning-based prognostic models

**Figure 2:**
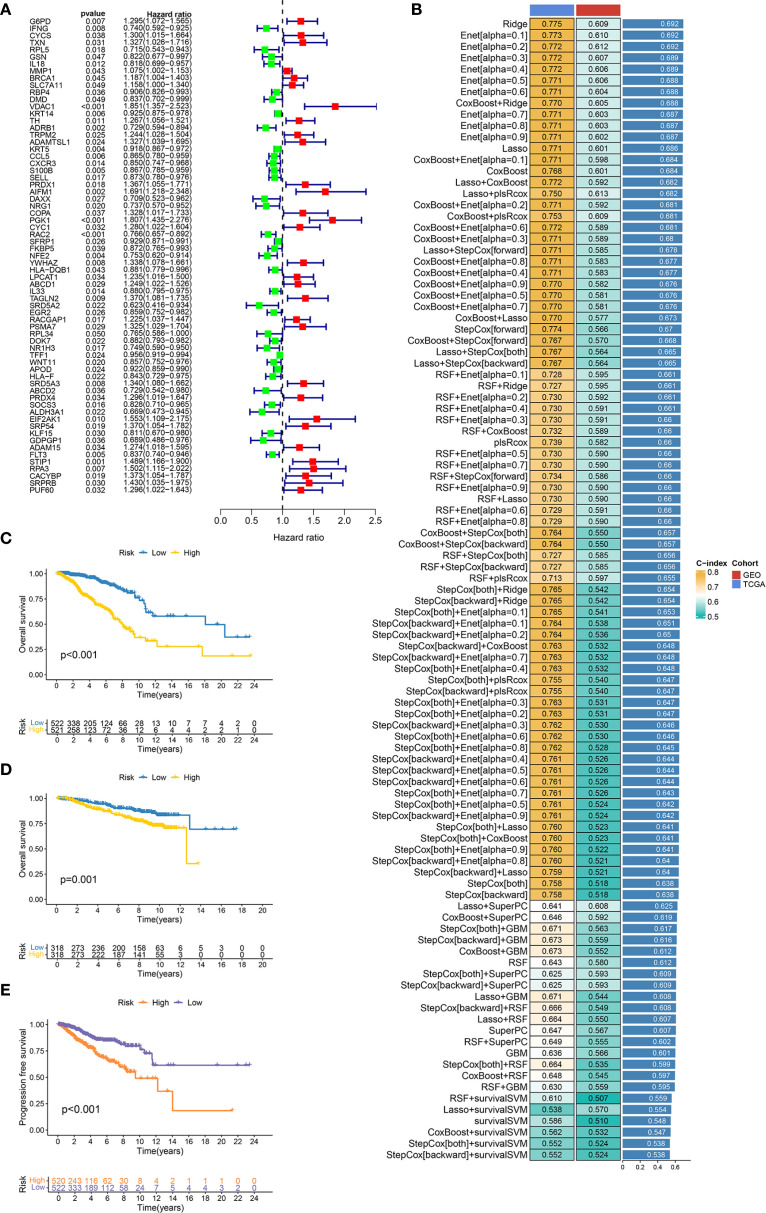
Construction and evaluation of machine learning prognostic models. **(A)** Forest plot displays the results of univariate Cox regression analysis for candidate prognostic DESORGs genes. **(B)** Bar chart comparing the performance (C-index) of 10 machine learning models and 101 feature selection combinations for predicting prognosis. Higher C-index values indicate better model performance. **(C, D)** Kaplan-Meier survival curve for overall survival in TCGA dataset **(C)** and GEO dataset **(D)**, comparing patients stratified into high and low risk groups based on the prognostic signature. **(E)** Kaplan-Meier survival curve for progression-free survival (PFS) in TCGA dataset.

Univariate Cox regression analysis (**Figure 2A**) revealed 67 DESORGs significantly linked to breast cancer prognosis, including 36 potentially protective genes (hazard ratio [HR] < 1), while 31 genes were associated with increased risk (HR > 1). To develop a robust prognostic model, we evaluated 101 models generated from combinations of 10 different machine learning algorithms (**Figure 2B**). The Ridge regression model outperformed other models, achieving greatest average C-index of 0.692 across all evaluated models. Specifically, it yielded a C-index of 0.775 in the TCGA training cohort and 0.609 in the GEO validation cohort, indicating consistent prognostic ability across different datasets. The Ridge model stratified TCGA patients into high/low-risk groups, with poorer survival in high-risk cases (*p* < 0.001; **Figure 2C**). This finding was consistently observed in the GEO validation cohort, where high-risk patients also had significantly worse OS (*p* = 0.001; **Figure 2D**). Furthermore, high-risk patients in testing cohort also exhibited shorter progression-free survival (PFS) (*p* < 0.001; **Figure 2E**). In conclusion, these results underscore the predictive capability of the Ridge regression model for both OS and PFS in breast cancer patients.

### 3.3 Interpretation of the optimal prognostic model using SHAP analysis

**Figure 3:**
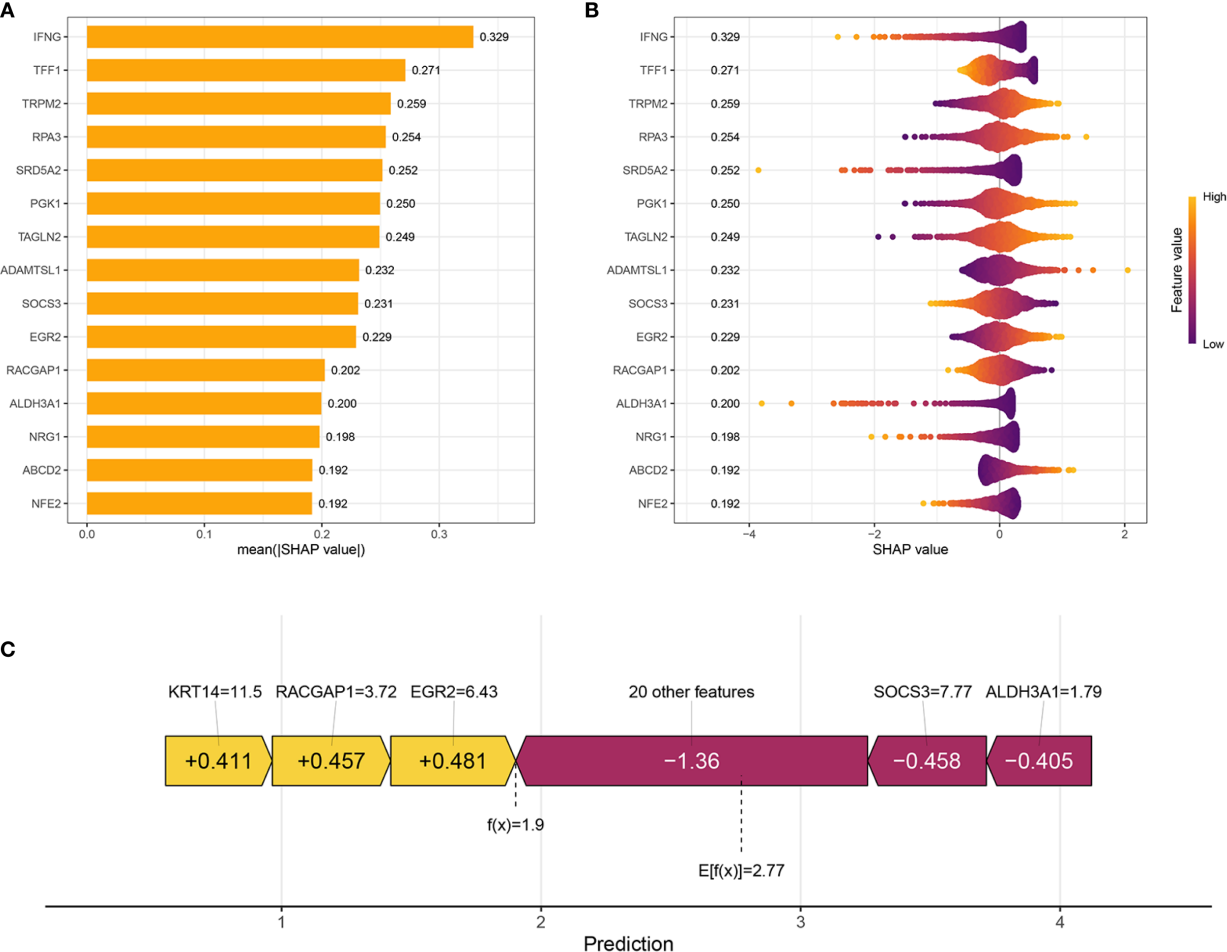
Interpretation of the prognostic model using SHAP Analysis. **(A)** Bar plot showing the global feature importance, ranked by the mean absolute SHAP value. Each bar represents a feature included in the prognostic model. **(B)** SHAP summary plot illustrates the distribution of SHAP values for each feature across all samples. Each dot represents a single sample for a given feature. **(C)** SHAP waterfall plot for an individual sample’s prediction, explaining how different features contribute to deviating the prediction from the base value.

To illustrate the interpretability of the Ridge model, SHAP values were used to explain feature importance and model predictions. The bar chart in **Figure 3A** illustrates the top-ranked features based on their mean absolute SHAP values, reflecting the average impact of each feature on the model’s predictions. Among these, IFNG is shown as the most important feature with a mean |SHAP value| of 0.329, followed by TFF1 (0.271), TRPM2 (0.259), RPA3 (0.254), SRD5A2 (0.252), and others. SHAP summary plot shown in **Figure 3B** provided a more detailed view of feature effects. Each row corresponds to a feature, ordered by global importance. Features where high values are predominantly associated with positive SHAP values include IFNG, TFF1, TRPM2, RPA3, SRD5A2, PGK1, TAGLN2, ADAMTSL1, EGR2, RACGAP1, NRG1, ABCD2, NFE2. Conversely, features like SOCS3 and ALDH3A1 show an opposite trend: high values are predominantly associated with negative SHAP values, indicating a tendency to decrease the model’s predicted outcome. SHAP waterfall plot (**Figure 3C**) shows how the model arrived at a prediction f(x) = 1.9 starting from a baseline expected value E[f(x)] = 2.77 (the average prediction over the dataset). Features contributing positively to the model’s prediction are highlighted in yellow with their corresponding positive SHAP values (e.g., KRT14 = 11.5 contributes +0.411, RACGAP1 = 3.72 contributes +0.457, EGR2 = 6.43 contributes +0.481). Features contributing negatively are shown in purple/maroon with their negative SHAP values (e.g., “20 other features” collectively contribute -1.36, SOCS3 = 7.77 contributes -0.458, ALDH3A1 = 1.79 contributes -0.405).

### 3.4 Development and validation of an integrated nomogram

**Figure 4:**
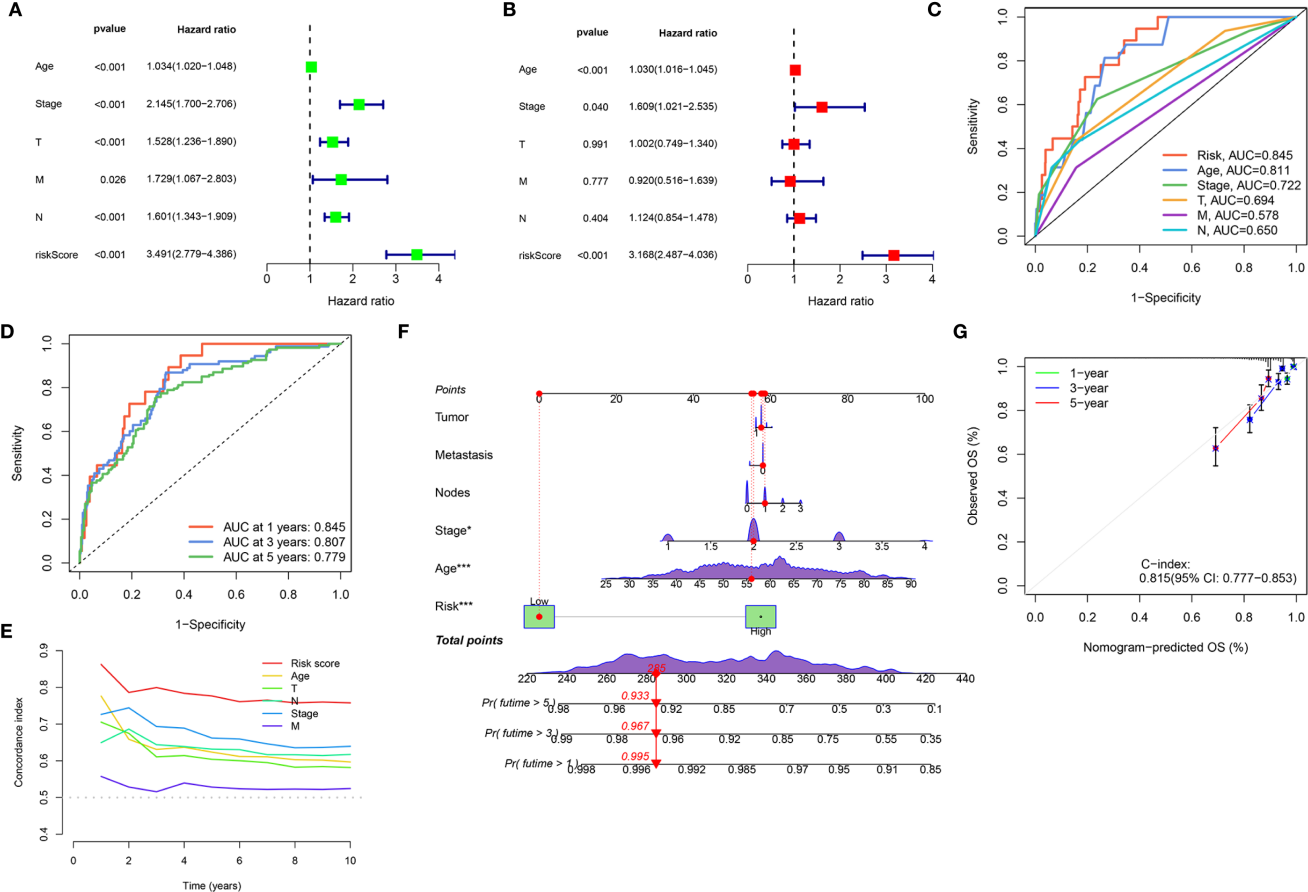
Development and validation of an integrated nomogram. **(A, B)** Forest plots display the results of univariate **(A)** and multivariate **(B)** Cox regression analyses for overall survival. **(C)** ROC curves evaluating the predictive accuracy of the Risk Score, Age, Stage, T, M, and N for overall survival. **(D)** Time-dependent ROC curves for the Risk Score, showing its predictive accuracy for overall survival at 1 year, 3 years, and 5 years. **(E)** C-index analysis over 10 years for the Risk Score compared to Age, Stage, T, M, and N. **(F)** Nomogram integrates clinical variables and the Risk Score for predicting 1-year, 3-year, and 5-year overall survival probability. **(G)** Calibration plot for the nomogram, assessing the agreement between nomogram-predicted overall survival (OS) and observed OS at 1-year, 3-year, and 5-year time points. (*P < 0.05, ***P < 0.001).

We aimed to create a practical clinical tool for overall survival (OS) prediction, and integrated the risk score derived from the Ridge regression model with relevant clinical variables from the TCGA dataset, including age, pathological stage, tumor size (T classification), lymph node (N classification), and metastasis (M classification). Univariate Cox proportional hazards regression analysis (**Figure 4A**) revealed that all considered variables were associated with increased risk, with the “riskScore” exhibiting the strongest association (HR = 3.491, *p* < 0.001). In the multivariate Cox regression analysis (**Figure 4B**), the “riskScore” remained a significant independent predictor of OS (HR = 3.168, *p* < 0.001), even after adjusting for other clinical factors. Age (HR = 1.030, *p* < 0.001) and pathological stage (HR = 1.609, *p* = 0.040) also were independent predictors. ROC analysis compared the ‘Risk’ model’s predictive accuracy against individual clinical variables, with AUC values calculated for quantitative evaluation (**Figure 4C**). The “Risk” model achieved a superior AUC of 0.845 compared to Age (0.611), Stage (0.722), T stage (0.631), M stage (0.578), and N stage (0.650), indicating its enhanced ability to discriminate between patients with different survival outcomes. Time-dependent ROC analyses further demonstrated the model’s consistent predictive performance over time, with AUC values of 0.845, 0.807, and 0.779 at 1, 3, and 5 years, respectively (**Figure 4D**). Additionally, the risk score consistently exhibited a higher C-index compared to traditional clinical variables across different time points, further supporting its superior prognostic value (**Figure 4E**). Furthermore, a nomogram was developed that integrates clinical variables with the risk score to predict OS probabilities (**Figure 4F**). To assess the nomogram’s accuracy, we constructed a calibration curve, which demonstrated satisfactory performance. The nomogram also exhibited good discriminative ability, with a C-index of 0.815 (95% CI: 0.777-0.853; **Figure 4G**).

### 3.5 Prognostic performance of the risk stratification model across diverse patient subgroups

**Figure 5:**
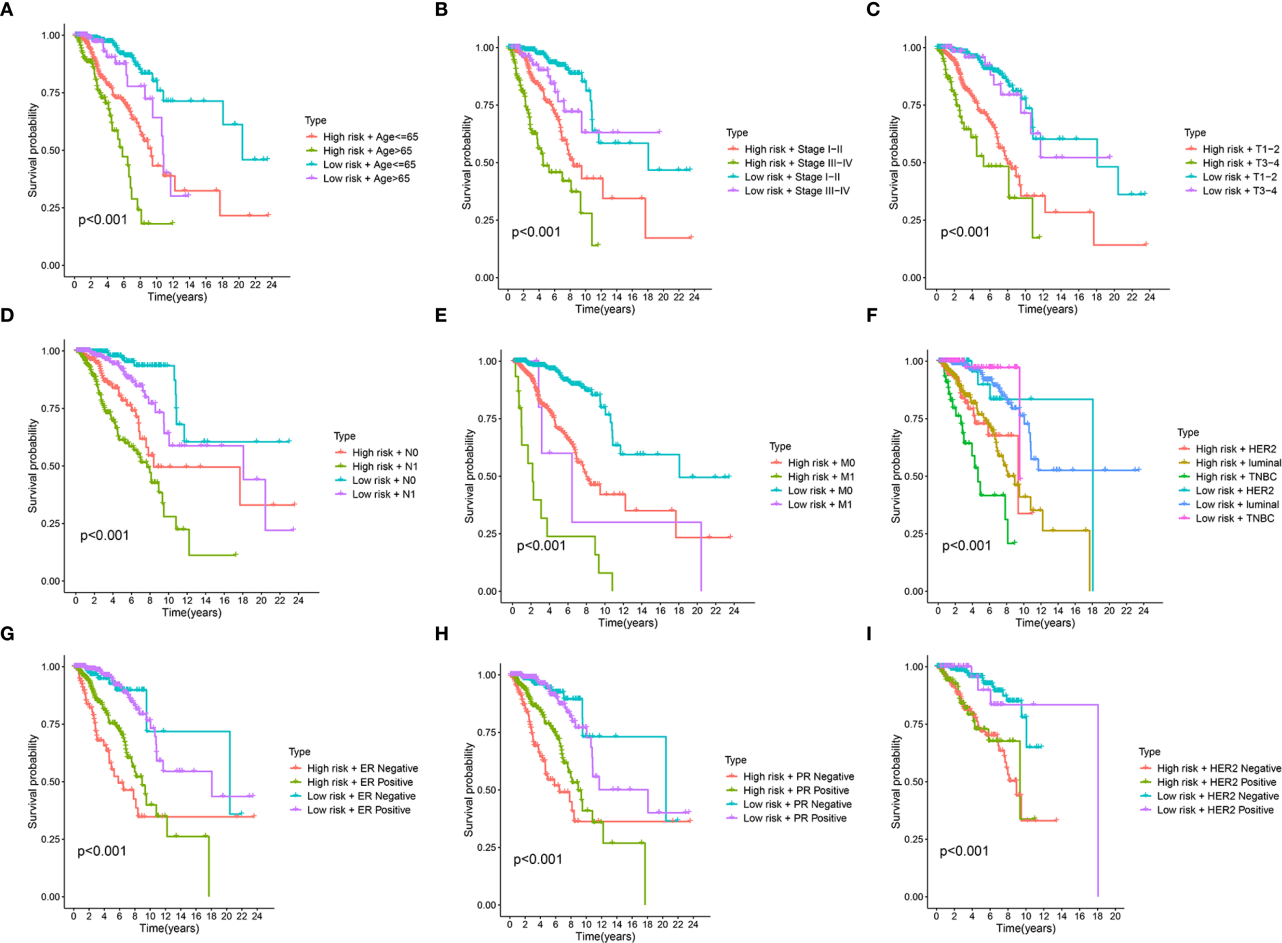
Stratified survival analysis of the prognostic riskscore across different clinical and molecular subgroups. **(A-I)** Kaplan-Meier survival curves illustrate the prognostic performance of the risk score in various patient subgroups. In each panel, patients are first divided into high-risk and low-risk groups based on the prognostic signature and then further stratified by Age **(A)**, clinical Stage **(B)**, T stage (tumor size) **(C)**, N stage (node) **(D)**, M stage (metastasis) **(E)**, molecular subtype (luminal, HER2, TNBC) **(F)**, Estrogen Receptor (ER) status **(G)**, Progesterone Receptor (PR) status **(H)**, Epidermal Growth Factor Receptor 2 (HER2) status **(I)**.

To assess the robustness of our risk stratification model, its prognostic power within various clinically defined subgroups were further evaluated. **Figures 5A-E** demonstrates the risk score’s robust prognostic value, with low-risk patients showing superior survival versus high-risk counterparts across all clinical strata (age, pathological stage, TNM classification; all p<0.001). Similarly, the risk score demonstrated consistent prognostic ability within molecularly defined subgroups, including molecular subtypes (Luminal, HER2, and TNBC), ER status, PR status, and HER2 status (all p < 0.001) (**Figures 5F-I**). These findings underscore the robust and independent prognostic value of our risk score across a wide spectrum of established clinical and molecular prognostic factors, consistently identifying patient groups with differing survival probabilities regardless of these other variables.

### 3.6 Mendelian randomization study between hub SORGs and breast cancer

**Figure 6:**
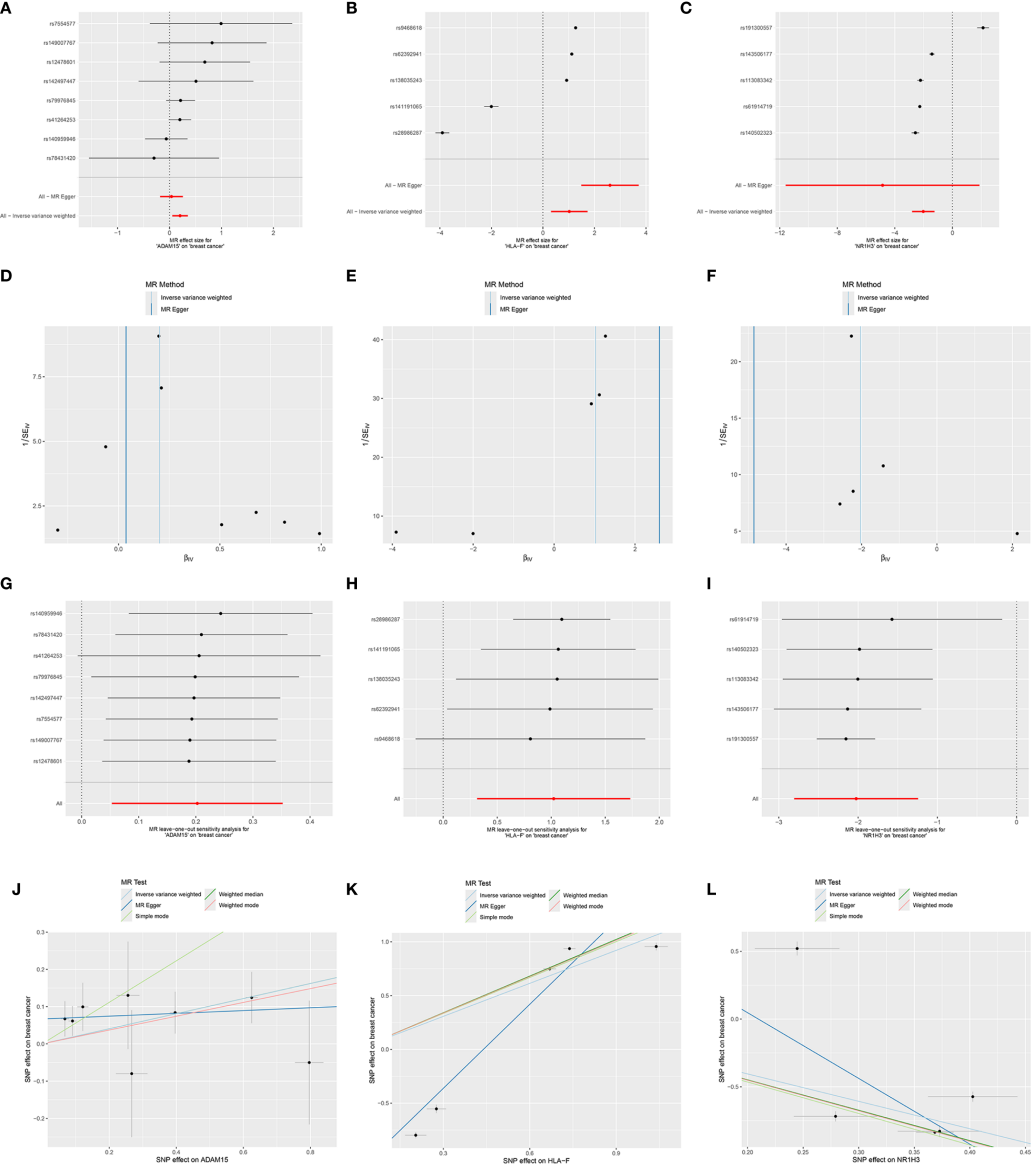
Mendelian randomization analyses for the causal effects of ADAM1S, HLA-F, and NR1H3 on breast cancer risk. **(A-C)** Forest plots showing the causal effect estimates of each individual Single Nucleotide Polymorphism (SNP) on breast cancer risk, mediated through the respective gene (ADAM1S, HLA-F, and NR1H3)’s expression. **(D-F)** Funnel plots visualizing the distribution of SNP effects on breast cancer (βGY​) against their precision (1/SEGY​). These plots are used to assess heterogeneity and potential directional pleiotropy. **(G-I)** Leave-one-out sensitivity analysis plots. Each point represents the MR estimate (IVW method) for the causal effect of the respective gene’s expression on breast cancer when the indicated SNP (y-axis) is excluded from the analysis. **(J-L)** Scatter plots illustrate the relationship between the SNP effects on the respective gene’s expression (x-axis) and the SNP effects on breast cancer risk (y-axis).

**Figure 7:**
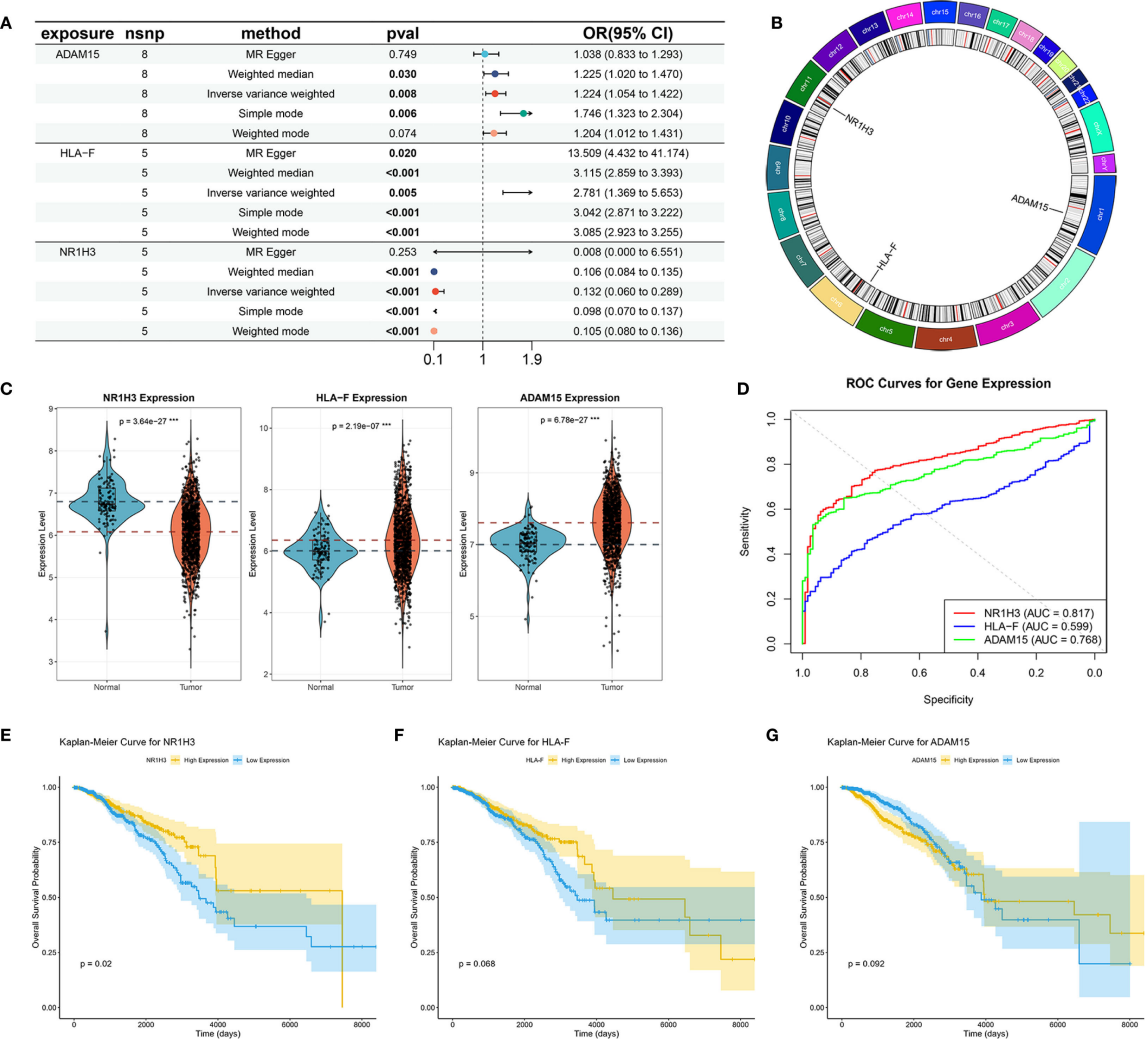
Mendelian Randomization, Expression Analysis, and Prognostic Significance of ADAM15, HLA-F, and NR1H3. **(A)** Summary of Mendelian Randomization (MR) results for the causal effects of ADAM15, HLA-F, and NR1H3 expression on breast cancer risk. **(B)** Circos plot illustrates the genomic locations and surrounding regions of the genes ADAM15, HLA-F, and NR1H3. **(C)** Violin plots comparing the expression levels of NR1H3, HLA-F, and ADAM15 between normal and tumor tissues. **(D)** ROC curves evaluating the performance of NR1H3, HLA-F and ADAM15 gene expression in distinguishing between normal and tumor. **(E-G)** Kaplan-Meier curves for overall survival based on the expression levels of NR1H3 **(E)**, HLA-F **(F)**, and ADAM15 **(G)**.

MR analysis was performed to assess causal links between the 67 prognostic DESORGs from the Ridge regression model and breast cancer risk. **Figures 6A-C** showed the estimated effect of each individual genetic variant (SNP, listed on the y-axis) used as an instrumental variable on breast cancer risk. In **Figures 6D-F**, these scatter plots where each point represents an instrumental SNP. The x-axis shows the SNP’s effect on breast cancer (β_XY), and the y-axis shows the inverse of the standard error of this effect (1/SE(β_XY)), indicating precision. Leave-One-Out sensitivity analysis (**Figures 6G-I**) shown how the overall MR estimate for the effect of ADAM15, HLA-F and NR1H3 on breast cancer changes by sequentially excluding each single nucleotide polymorphism (SNP). **Figures 6J-L** displayed the relationship between each SNP’s effect on the exposure (e.g., “SNP effect on ADAM15”) and its corresponding effect on breast cancer risk. Various MR methods were employed (**Figure 7A**). Weighted median, IVW and Simple mode methods show ADAM15 has a statistically significant odds ratio. All MR methods indicate HLA-F has a strong and statistically significant causal risk effect, with ORs substantially greater than 1 (e.g., IVW OR = 3.115, 95% CI: 2.859-3.393; Weighted mode OR = 2.781, 95% CI: 1.366-5.659). Most MR methods suggest NR1H3 has a strong and statistically significant protective causal effect, with ORs substantially less than 1 (e.g., IVW OR = 0.132, 95% CI: 0.060-0.289; Weighted mode OR = 0.105, 95% CI: 0.080-0.136). A circos plot designed to visualize genomic information, including the chromosomal locations of genes (**Figure 7B**): ADAM15 is located on chromosome 1, HLA-F is on chromosome 6, and NR1H3 is on chromosome 11. Compared to normal tissues, NR1H3 expression is significantly downregulated in tumor samples, whereas HLA-F and ADAM15 expression are significantly higher in tumor tissues (**Figure 7C**). ROC curves were generated to evaluate the ability of each gene to discriminate tumor and normal tissues. NR1H3 has the highest AUC of 0.817, ADAM15 has an AUC of 0.768, HLA-F has an AUC of 0.598 (**Figure 7D**), suggesting NR1H3 expression has the best discriminatory power among the three for distinguishing tumor from normal tissue. Kaplan-Meier survival analyses showed that only NR1H3 is associated with OS (*p* = 0.02) (**Figure 7E**). Higher expression of NR1H3 predicted a significantly better OS. Although lower HLA-F expression showed a trend toward better OS, this did not reach statistical significance (*p* = 0.068) (**Figure 7F**). No significant association was observed between ADAM15 expression and OS (p = 0.092) (**Figure 7G**).

### 3.7 NR1H3 suppresses proliferation and metastasis

**Figure 8:**
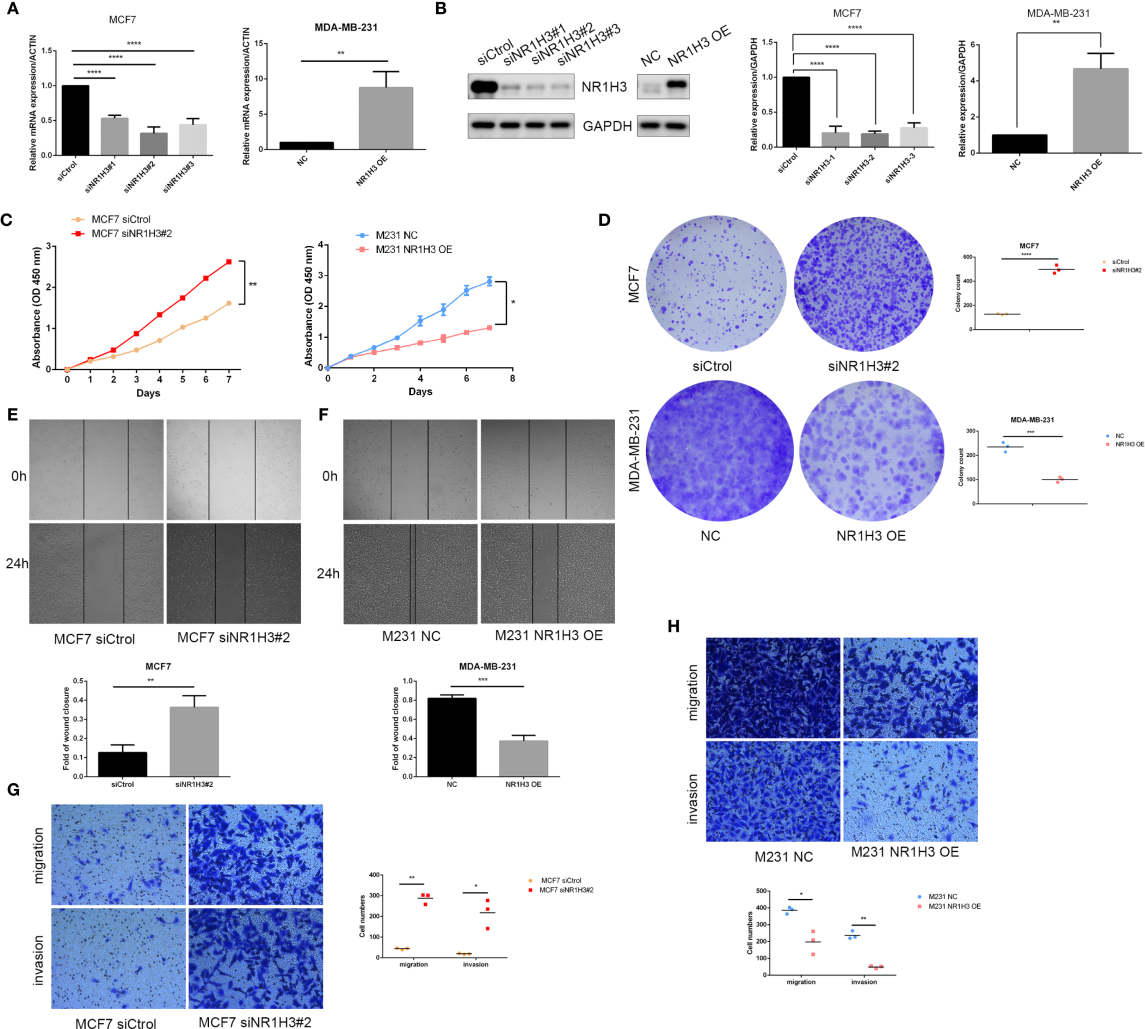
Functional effects of NR1H3 modulation on breast cancer cell proliferation, colony formation, migration, and invasion *in vitro*. **(A, B)** Verification of NR1H3 mRNA level and protein level in MCF7 and MDA-MB-231 cells by qRT-PCR **(A)** and western blot **(B)**, respectively. **(C)** Cell proliferation assays (absorbance at OD 450 nm) over 7 days for MCF7 cells treated with siCtrol or siNR1H3#2, and for MDA-MB-231 cells with NC or NR1H3 OE. **(D)** Colony formation assays. Representative images and quantification of colony counts for MCF7 and MDA-MB-231 cells. **(E, F)** Wound healing assays for MCF7 **(E)** and MDA-MB-231 **(F)** cells. Representative images at 0h and 24h after scratching and quantification of the fold of wound closure. **(G, H)** Transwell migration and invasion assays for MCF7 **(G)** and MDA-MB-231 **(H)** cells. Representative images of migrated and invaded cells and quantification of cell numbers. (*P < 0.05, **P < 0.01, ***P < 0.001).

To further explore the function of NR1H3 expression in breast cancer cell lines, we overexpressed NR1H3 in MDA-MB-231 cells and knockdown NR1H3 in MCF7 cells. The mRNA and protein levels were verified by qRT-PCR and western blot, respectively (**Figures 8A, B**). siNR1H3#2 exhibiting the best knockdown efficiency was selected for further experiments. Knockdown of NR1H3 (siNR1H3#2) in MCF7 cells significantly increased cell proliferation over 7 days compared to the control, while overexpressed NR1H3 in MDA-MB-231 had opposite effect (**Figure 8C**). Similarly, reducing NR1H3 expression increased the number of colonies formed in MCF7 cells, overexpression of NR1H3 in MDA-MB-231 cells resulted in a marked reduction in colony formation (**Figure 8D**). The wound healing assay demonstrated that NR1H3 downregulation increases while NR1H3 upregulation decreases the rate of wound closure (cell migration) at 24 hours compared to the control group (**Figure 8E**). Consistently, NR1H3 siRNA#2 significantly increased both cell migration and invasion through transwell membrane. In contrast, NR1H3 overexpression led to a substantial decrease in these invasive behaviors (**Figure 8F**). Collectively, these findings suggest that NR1H3 functions as a tumor suppressor in breast cancer by inhibiting cell proliferation, colony formation, migration and invasion.

### 3.8 GSEA and GSVA analyses

**Figure 9:**
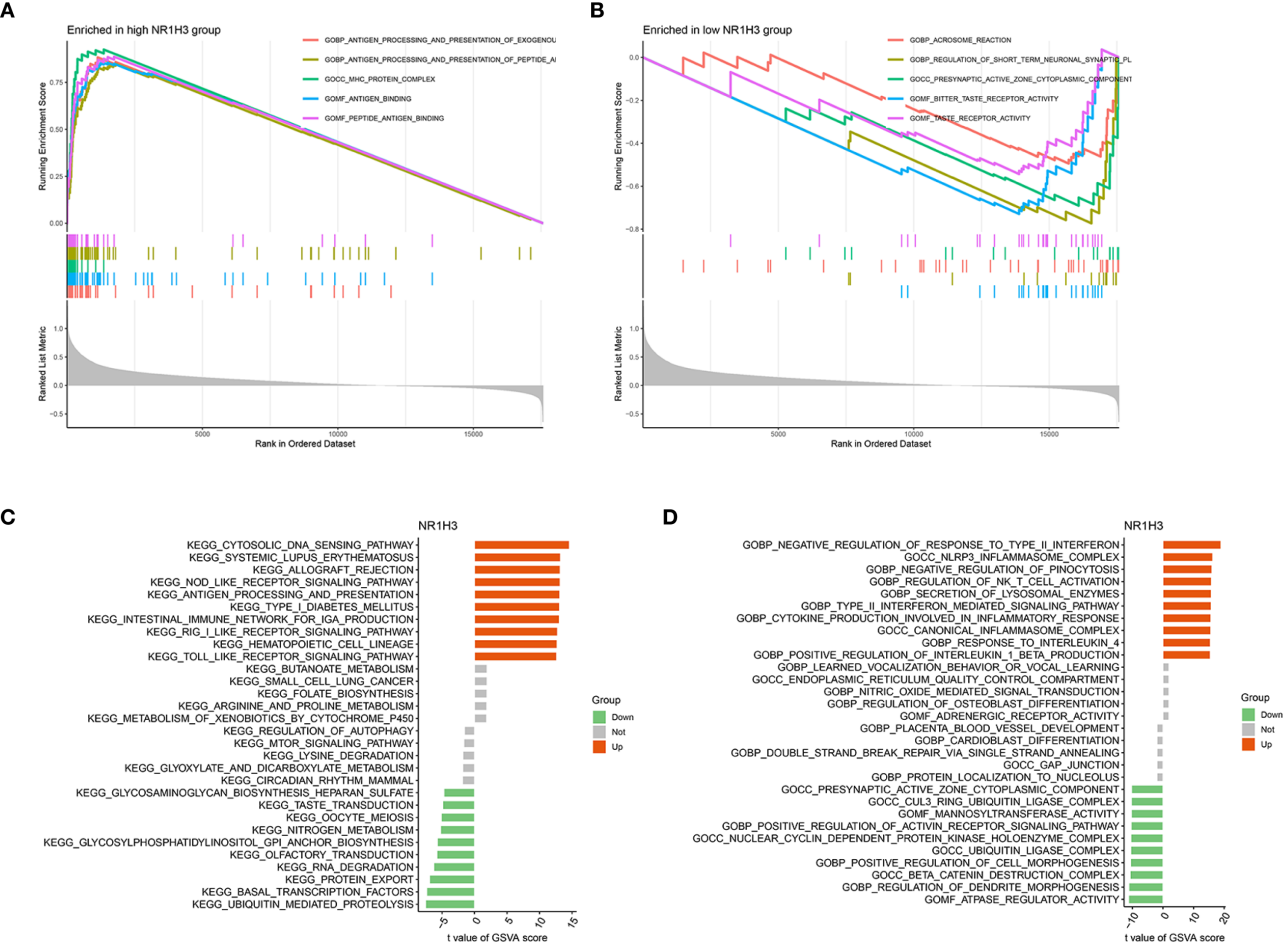
Gene Set Enrichment Analysis (GSEA) and Gene Set Variation Analysis (GSVA) reveal pathways associated with NR1H3 Expression. **(A)** GSEA plot showing top gene sets enriched in the high NR1H3 expression group. **(B)** GSEA plot illustrating top gene sets enriched in the low NR1H3 expression group. **(C)** Bar plot displaying significantly altered KEGG pathways identified by GSVA in relation to NR1H3 expression. **(D)** Bar plot showing significantly altered Gene Ontology (GO) terms identified by GSVA in relation to NR1H3 expression.

To explore the pathways that NR1H3 involved in breast cancer, GSEA analysis was conducted. Among pathways enriched in samples with high NR1H3 expression, the top five were primarily linked to immune responses: GOBP_ANTIGEN_PROCESSING_AND_PRESENTATION_OF _EXOGENOUS_ ANTIGEN, GOCC_ MHC_PROTEIN_COMPLEX, GOMF_ANTIGEN_BINDING and GOMF_PEPTIDE_ANTIGEN_BINDING (**Figure 9A**). The enriched pathways in low NR1H3 group were more diverse and include: GOBP_AEROBIC_RESPIRATION, GOCC_PRESYNAPTIC_ACTIVE_ZONE_CYTOPLASMIC_ COMPONENT, GOBP_SENSORY_PERCEPTION_OF_TASTE and GOMF_TASTE_RECEPTOR_ ACTIVITY, which indicates that low NR1H3 levels are associated with alterations in cellular respiration and some neuronal or sensory-related pathways (**Figure 9B**). GSVA for KEGG pathways showed their correlation with NR1H3 expression (indicated by t-values). Pathways positively correlated with NR1H3 are again heavily involved in immune processes, such as: KEGG_CYTOSOLIC_DNA_SENSING_PATHWAY, KEGG_SYSTEMIC_LUPUS_ERYTHEMATOSUS, and KEGG_ANTIGEN_PROCESSING_AND_PRESENTATION, while pathways negatively correlated with NR1H3 include processes like: KEGG_PROTEIN_ EXPORT and KEGG_UBIQUITIN_ MEDIATED_PROTEOLYSIS (**Figure 9C**). Similarly, GSVA for GO pathways show that pathways positively correlated with NR1H3 further confirm the strong association with immune activation, while pathways negatively correlated with NR1H3 are related to chromosomal organization and morphogenesis (**Figure 9D**).

### 3.9 Molecular docking of NR1H3 and related ingredients

**Figure 10:**
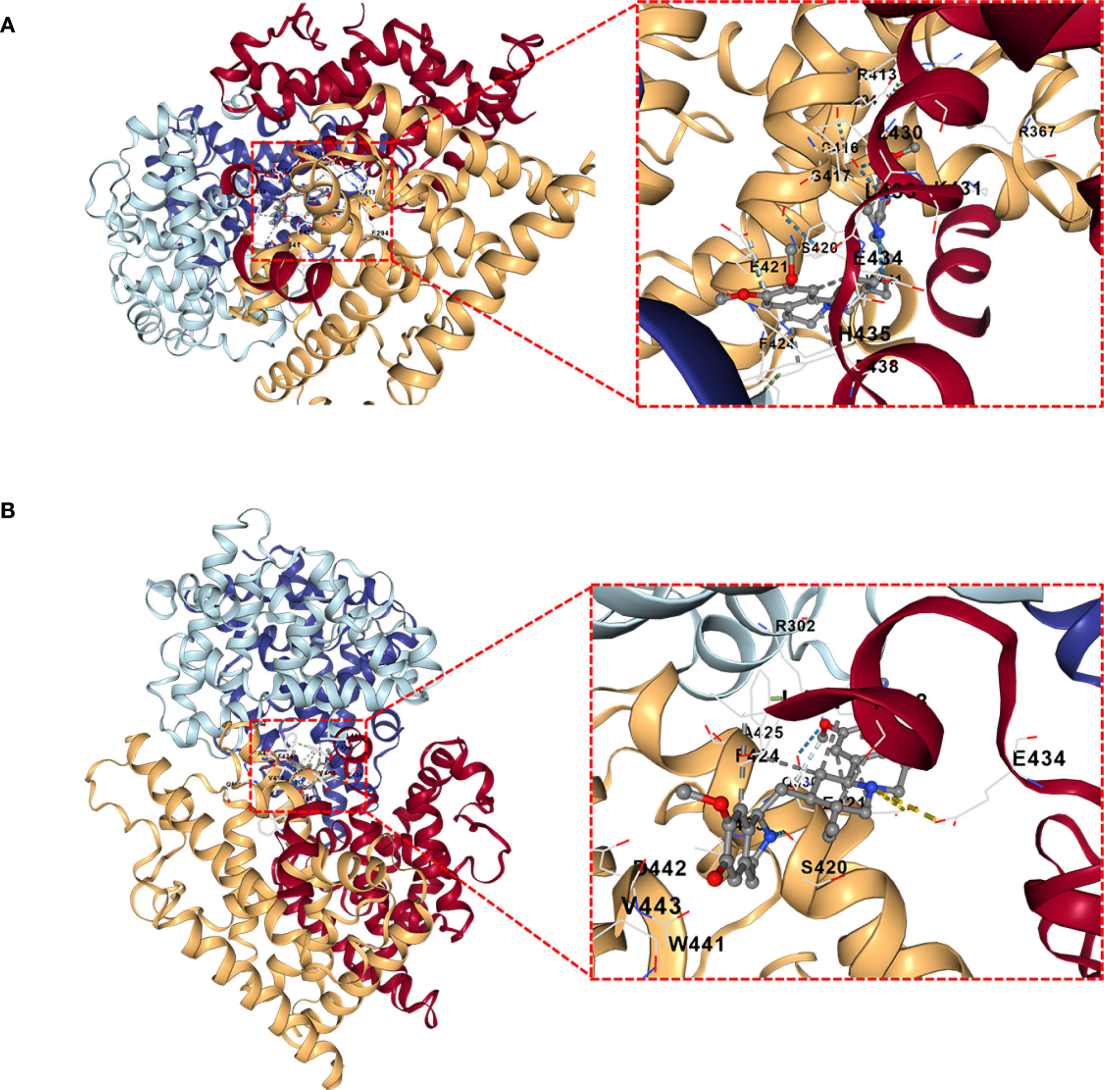
Molecular docking interactions of Cephaeline and Emetine with NR1H3 Protein. **(A, B)** Overall view of NR1H3- Cephaeline complex **(A)** and NR1H3- Emetine complex **(B)** with a magnified insect showing the detailed interactions at the binding site. Specific amino acid residues of the protein are depicted interacting with the bound ligand. Dashed lines likely indicate hydrogen bonds or other key interactions.

DSigDB_All_detailed data was downloaded from DSigDB database. 155 drugs were identified associated with NR1H3. Among them, Cephaeline and Emetine were identified as potential upregulators of NR1H3 via the Connectivity Map (CMAP) database. This database contains gene expression data from cell lines treated with various compounds. CMAP data suggested that both Cephaeline and Emetine treatment lead to an upregulation of NR1H3 expression in MCF7 and HL60 cells. Following this lead from the gene expression data, we then performed molecular docking to investigate a plausible mechanism. Molecular docking shows that there are five potential binding pockets (C1 through C5) for the drug Cephaeline on the NR1H3 protein. The predicted binding affinities (Vina scores) for Cephaeline range from -9.2 kcal/mol (strongest binding, pocket C1) to -7.1 kcal/mol (weakest among those listed, pocket C5) (**Table 1**). The binding pocket C1 was shown in **Figure 10A** displays visualizations of protein-ligand interactions between NR1H3 and Cephaeline. The ligand is shown interacting with 39 specific amino acid residues of NR1H3 protein in chain A, chain B and chain C. There are also five potential binding pockets (C1 through C5) for the drug Emetine on the NR1H3 protein. The predicted binding affinities (Vina scores) for Emetine range from -9.1 kcal/mol (strongest, pocket C1) to -7.3 kcal/mol (pocket C3) (**Table 2**). The ligand is shown interacting with 31 specific amino acid residues of NR1H3 protein in chain A, chain B and chain C (**Figure 10B**). The favorable binding affinities (Vina scores of -9.2 kcal/mol for Cephaeline and -9.1 kcal/mol for Emetine) suggest a strong and stable interaction is possible.

**Table 1:** Structure-based blind docking of NR1H3 with cephaeline.

Curpocket ID	Vina score	Cavity volume (Å^3^)	Center (x, y, z)	Docking size (x, y, z)	Contact residues
C1	-9.2	3735	69, 56, 24	24, 31, 24	**Chain A**: ARG426 SER427 LEU430 LYS431 GLU434 HIS435 PHE438 PHE439 LEU441 **Chain B**: LYS289 THR290 ILE293 GLU294 GLN330 ARG367 PRO368 ASN369 LEU412 ARG413 THR414 SER416 SER417 HIS419 SER420 GLU421 VAL423 PHE424 ALA425 ARG427 LEU428 ILE440 TRP441 ASP442 VAL443 **Chain C**: GLN270 ASP273 LEU276 ARG302 TRP305
C2	-8.9	1770	89, 38, 10	24, 24, 24	**Chain B**: GLN330 GLU332 **Chain C**: ILE299 ARG302 ALA303 GLY304 TRP305 ASN306 GLU307 ASN377 PRO378 ASP379 SER380 LYS381 GLY382 LEU383 PRO386 ALA387 GLU390 ARG393 GLU394 TYR397 ARG426 GLY429 LEU430 LEU433 **Chain D**: GLU332 ASN335 PRO336 GLU339 PHE340 ARG342 ALA343 GLU346 LEU347 PRO403 ARG404 MET405 MET407 LYS408 SER411 THR414 LEU415 SER417 VAL418 GLU421
C4	-8.5	1557	74, 42, 35	24, 24, 24	**Chain A**: ILE299 ARG302 ALA303 TRP305 ASN306 GLU307 ASN377 PRO378 ASP379 SER380 LYS381 GLY382 LEU383 PRO386 ALA387 GLU390 ARG393 TYR397 ARG426 GLY429 LEU430 LEU433 GLU434 **Chain B**: GLU332 ASN335 PRO336 GLU339 PHE340 SER341 ARG342 ALA343 GLU346 LEU347 PRO403 ARG404 MET405 MET407 LYS408 SER411 THR414 LEU415 SER417 VAL418 SER420 GLU421 **Chain D**: GLU332
C3	-7.8	1578	90, 58, 13	24, 24, 24	**Chain A**: LEU436 PHE439 LYS440 Chain C: ARG334 HIS338 ALA340 GLY341 VAL342 GLY343 ALA344 ILE345 ASP347 ARG348 LEU350 THR351 GLU352 SER427 LEU430 LYS431 GLU434 **Chain D**: LYS289 THR290 ILE293 GLU294 ALA365 ASP366 ARG367 PRO368 ASN369 VAL370 GLN371 GLN373 ARG413 SER416 SER417 HIS419 SER420 ILE440 TRP441 ASP442 VAL443
C5	-7.1	1516	55, 40, 24	24, 24, 24	**Chain B**: VAL216 GLN219 GLN220 GLN221 ASN223 ARG224 SER226 PHE227 PHE252 PHE255 THR256 LEU258 ALA259 VAL261 SER262 GLU265 ILE266 ILE293 MET296 LEU297 GLU299 THR300 ARG302 ARG303 TYR304 ASN305 ILE311 THR312 PHE313 LEU314 LYS315 SER318 PHE324 LEU329 PHE333 ILE334 ILE337 PHE338 SER341 ASP351 HIS419 GLN422 LEU426 LEU433 LEU437 TRP441

**Table 2:** Structure-based Blind Docking of NR1H3 with Emetine.

Curpocket ID	Vina score	Cavity volume (Å^3^)	Center (x, y, z)	Docking size (x, y, z)	Contact residues
C1	-9.1	3735	69, 56, 24	25, 31, 25	**Chain A**: ARG348 SER427 LEU430 LYS431 LEU433 GLU434 HIS435 PHE438 PHE439 LEU441 **Chain B**: LYS289 THR290 ILE293 GLU294 ARG367 PRO368 ASN369 ARG413 SER416 SER417 HIS419 SER420 GLU421 VAL423 PHE424 ARG427 ILE440 TRP441 ASP442 VAL443 **Chain C**: ASP273
C2	-9.0	1770	89, 38, 10	25, 25, 25	**Chain A**: HIS435 **Chain B**: GLN330 GLU332 LEU428 **Chain C**: ILE299 ARG302 ALA303 GLY304 TRP305 ASN306 GLU307 ASN377 PRO378 ASP379 SER380 LYS381 GLY382 PRO386 ALA387 GLU390 ARG393 GLU394 TYR397 ARG426 GLY429 LEU430 LEU433 GLU434 **Chain D**: GLU332 PRO336 GLU339 PHE340 ARG342 ALA343 MET344 GLU346 LEU347 ARG404 MET407 LYS408 VAL410 SER411 THR414 LEU415 SER417 VAL418 GLU421
C3	-7.3	1578	90, 58, 13	25, 25, 25	**Chain A**: ASP263 **Chain C**: ARG334 HIS338 GLY341 VAL342 GLY343 ALA344 ILE345 ASP347 ARG348 LEU350 THR351 GLU352 SER427 LEU430 LYS431 GLU434 **Chain D**: LYS289 THR290 ILE293 GLU294 ALA365 ASP366 ARG367 PRO368 ASN369 VAL370 GLN371 GLN373 ARG413 SER416 SER417 HIS419 SER420 ILE440 TRP441
C4	-8.3	1557	74, 42, 35	25, 25, 25	**Chain A**: ILE299 ARG302 ALA303 GLY304 TRP305 ASN306 GLU307 ASN377 PRO378 ASP379 SER380 LYS381 PRO386 ALA387 GLU390 ARG426 GLY429 LEU430 LEU433 **Chain B**: GLU332 ASN335 PRO336 GLU339 PHE340 ARG342 ALA343 GLU346 LEU347 ARG404 LYS408 SER411 THR414 LEU415 SER417 VAL418 GLU421 **Chain D**: GLU332
C5	-7.6	1516	55, 40, 24	25, 25, 25	**Chain B**: GLN219 GLN220 ASN223 ARG224 SER226 PHE227 PHE252 PHE255 THR256 LEU258 ALA259 VAL261 SER262 GLU265 MET296 LEU297 GLU299 THR300 ARG302 ARG303 TYR304 ASN305 PRO306 THR312 PHE313 LEU314 LYS315 SER318 PHE324 LEU329 ASN345 ASP351 LEU426 LEU433 LEU437 TRP441

## 4 Discussion

Using machine learning, we established a new prognostic signature derived from differentially expressed sodium overload-related genes (DESORGs), with thorough development and validation. Among these, we identified NR1H3 as a key gene and experimentally confirmed its tumor-suppressive function in breast cancer cells. Our initial analysis of the GeneCards database yielded 2052 sodium overload-related genes, of which 753 showed differential expression in comparison between normal and tumor samples. Notably, the most significantly downregulated genes included VEGFD, CAVIN2, CAV1, and ADRB2, while CDKN3, INHBA, AURKB, MMP13, and EZH2 were significantly upregulated. Caveolin-1 (CAV1) has been reported to play dual roles in breast cancer, acting as both a tumor suppressor and promoter depending on the specific cellular context and breast cancer subtype (20). Adrenergic Receptor Beta 2 (ADRB2) has been implicated in cancer cell proliferation and stress responses (21). Conversely, the upregulation of genes such as Aurora Kinase B (AURKB) and Enhancer of Zeste Homolog 2 (EZH2) is commonly associated with increased cell proliferation and poor prognosis in breast cancer (22, 23).

Pathway enrichment analyses provided valuable insights into the functional consequences of the observed DESORG expression changes. KEGG analysis revealed significant enrichment in pathways critical for cancer development and progression, including PI3K-Akt and MAPK signaling pathway, lipid and atherosclerosis, and calcium signaling pathway. The PI3K-Akt and MAPK signaling pathways are well-known drivers of cancer cell growth, survival, and proliferation (24, 25). Given the interconnectedness of sodium and calcium transport through mechanisms like Na+/Ca2+ exchangers (26), alterations in DESORGs could directly impact calcium signaling within tumor cells. Leveraging these differentially expressed sodium overload-related genes, we identified 67 genes with significant prognostic value using univariate Cox regression. We then constructed and evaluated 101 prognostic models using ten different machine learning algorithms. Among these, the Ridge regression model emerged as the optimal model. This validation across independent datasets underscores the reliability and generalizability of the prognostic signature (27). Using the prognostic model, we stratified patients into high- and low-risk categories, which showed markedly distinct OS and PFS outcomes. Specifically, high-risk patients experienced substantially worse outcomes (*p* < 0.001 in TCGA, *p* = 0.001 in GEO for OS). The model’s ability to significantly stratify patients underscores its clinical potential.

Integrating the DESORG-derived risk score with established clinical variables— such as age, tumor stage, and TNM classification—into a nomogram significantly improved prognostic accuracy. The risk score emerged as a strong independent predictor and risk factors for BC patients (multivariate HR: 3.168, 95% CI:2.487-4.036), outperforming individual clinical factors in AUC analysis (Risk model AUC: 0.845). The novel nomogram demonstrated strong predictive accuracy (C-index = 0.815), indicating its clinical utility. Such integrated models can facilitate more personalized risk assessment, thereby aiding in treatment decisions. Traditional prognostic markers in breast cancer, including hormone receptor status and TNM classification are widely utilized (28). However, their predictive power can be limited within specific subtypes, highlighting the need for more universally applicable biomarkers. Importantly, the prognostic effectiveness of our DESORG-based risk score was consistently observed across diverse clinically and molecularly defined patient subgroups. Regardless of stratification by age, tumor stage, T/N/M stages, molecular subtype (Luminal, HER2, TNBC), or ER/PR/HER2 status, the model consistently distinguished between high- and low-risk groups, with significant differences in survival outcomes. This consistent performance across heterogeneous subgroups emphasizes the fundamental function of DESORG-related biology in BC prognosis and suggests the broad applicability of the model in personalized risk assessment and treatment decision-making.

Our MR analysis explores potential causal relationships between expression level of key sodium overload-related genes and breast cancer risk. This analysis identified HLA-F as a significant causal risk factor (IVW OR = 3.115, p<0.001) and NR1H3 as a strong protective factor (IVW OR = 0.132, p<0.001) for breast cancer. ADAM15 also showed a statistically significant odds ratio with some MR methods. HLA-F has been shown to be upregulated in tumors and is associated with immune evasion and poor prognosis in various cancers (29, 30). The strong causal risk effect we identified for HLA-F warrants further investigation into its specific role in breast cancer pathogenesis linked to sodium overload pathways. Our MR analysis strongly suggested a protective role for NR1H3. Consistent with this finding, NR1H3 expression was significantly lower in BC tumors. More importantly, its low expression level predicts worse prognosis. NR1H3, also known as Liver X Receptor Alpha (LXRα), is a nuclear receptor involved in cholesterol homeostasis, lipid metabolism, and inflammation (31, 32). In breast cancer models, LXR has been shown to inhibit cell growth though effecting EST expression (33). In our study, *in vitro* experiments robustly confirmed the tumor-suppressive function of NR1H3 in breast cancer cell lines. NR1H3 gain-of-function attenuated oncogenic behaviors (proliferation, colony formation, motility), while loss-of-function in MCF7 cells exacerbated these phenotypes. These results align with previous studies indicating that LXRs can suppress breast cancer cell growth and metastasis (34, 35). GSEA and GSVA further elucidated the pathways associated with NR1H3 expression. High NR1H3 expression was strongly correlated with immune response pathways, including antigen processing and presentation (via both GO and KEGG), as well as cytosolic DNA sensing. This suggests that part of NR1H3’s protective effect may be mediated through the enhancement of anti-tumor immunity.

Molecular docking studies further predicted strong binding affinities of these compounds to NR1H3, suggesting potential therapeutic interactions. Emetine, an established anti-protozoal drug, has been demonstrated anti-cancer effects in various cancers, including gastric cancer (36). Cephaeline also inhibits cell viability and migration in Mucoepidermoid carcinoma (MEC) (37). However, their specific mechanism of action via NR1H3 in breast cancer requires further validation. It is important to clarify the distinction between the broad set of sodium overload-related genes (SORGs) used to build our prognostic model and the specific subset of genes that are mechanistic drivers of necrosis by sodium overload (NECSO). Our study intentionally cast a wide net, analyzing a comprehensive list of SORGs to build a robust prognostic signature for breast cancer. NECSO, however, is a specific form of regulated cell death defined by strict criteria, including persistent activation of ion channels like TRPM4 [8], a resulting massive influx of sodium, subsequent cell swelling, and eventual necrotic membrane rupture. While our machine learning model identified genes such as TRPM2 as having high prognostic importance, this statistical association does not automatically classify it as a direct NECSO mediator. To be confirmed as a true NECSO driver, TRPM2 would require dedicated functional validation to demonstrate its direct role in inducing these characteristic cellular events. Therefore, a critical direction for future research will be to functionally screen our list of prognostically significant DESORGs to determine which, if any, are bona fide mediators of NECSO in breast cancer. Such work would bridge our prognostic findings with the specific mechanisms of this novel cell death pathway.

## 5 Conclusion

This research developed and evaluated a novel DESORGs prognostic signature based on machine learning which shows significant potential for predicting BC patients’ survival. MR analysis provided causal insights into the roles of NR1H3 in breast cancer risk. Importantly, we experimentally validated NR1H3 as a tumor suppressor in breast cancer cells, influencing proliferation, colony formation, migration, and invasion. These findings highlight a novel link between sodium homeostasis, immune response, and breast cancer prognosis, offering new avenues for therapeutic intervention, potentially through the modulation of NR1H3 activity. Further investigation into these DESORGs may uncover novel mechanisms and treatment strategies for BC.

## 6 Limitations

Despite the robust findings of this study, several limitations should be acknowledged. First, our prognostic model was developed and validated using retrospective data from public repositories (TCGA and GEO). Although we demonstrated the model’s consistency across these datasets, its predictive power must be confirmed in prospective, multi-center clinical cohorts before clinical application. Second, the use of different transcriptomic platforms for the training (TCGA, RNA-seq) and validation (GEO, microarray) cohorts could introduce technical variability and potential batch effects. Third, our analyses relied on bulk tissue transcriptomic data, which provides an average of gene expression across all cell types within the tumor microenvironment. This approach may obscure cell-type-specific functions and interactions, a particularly relevant point given the strong link we identified between high NR1H3 expression and immune activation pathways.

## Data Availability

The original contributions presented in the study are included in the article/supplementary material. Further inquiries can be directed to the corresponding authors.

## References

[1] Bray F Laversanne M Sung H Ferlay J Siegel RL Soerjomataram I. Global cancer statistics 2022: globocan estimates of incidence and mortality worldwide for 36 cancers in 185 countries. CA Cancer J Clin. (2024) 74:229–63. doi: 10.3322/caac.21834, PMID: 38572751

[2] Łukasiewicz S Czeczelewski M Forma A Baj J Sitarz R Stanislawek A. Breast cancer-epidemiology, risk factors, classification, prognostic markers, and current treatment strategies-an updated review. Cancers (Basel). (2021) 13(17):4287. doi: 10.3390/cancers13174287, PMID: 34503097 PMC8428369

[3] Coleman MP Quaresma M Berrino F Lutz JM De Angelis R Capocaccia R. Cancer survival in five continents: A worldwide population-based study (Concord). Lancet Oncol. (2008) 9:730–56. doi: 10.1016/S1470-2045(08)70179-7, PMID: 18639491

[4] Pfeffer CM Singh ATK. Apoptosis: A target for anticancer therapy. Int J Mol Sci. (2018) 19. doi: 10.3390/ijms19020448, PMID: 29393886 PMC5855670

[5] Dai E Chen X Linkermann A Jiang X Kang R Kagan VE. A guideline on the molecular ecosystem regulating ferroptosis. Nat Cell Biol. (2024) 26:1447–57. doi: 10.1038/s41556-024-01360-8, PMID: 38424270 PMC11650678

[6] Xie J Yang Y Gao Y He J. Cuproptosis: mechanisms and links with cancers. Mol Cancer. (2023) 22:46. doi: 10.1186/s12943-023-01732-y, PMID: 36882769 PMC9990368

[7] Zou Z Zhao M Yang Y Xie Y Li Z Zhou L. The role of pyroptosis in hepatocellular carcinoma. Cell Oncol (Dordr). (2023) 46:811–23. doi: 10.1007/s13402-023-00787-9, PMID: 36864264 PMC12974679

[8] Fu W Wang J Li T Qiao Y Zhang Z Zhang X. Persistent activation of trpm4 triggers necrotic cell death characterized by sodium overload. Nat Chem Biol. (2025) 21(8):1238–49. doi: 10.1038/s41589-025-01841-3, PMID: 39915626

[9] Koike T Tanaka S Oda T Ninomiya T. Sodium overload through voltage-dependent na(+) channels induces necrosis and apoptosis of rat superior cervical ganglion cells *in vitro* . Brain Res Bull. (2000) 51:345–55. doi: 10.1016/s0361-9230(99)00246-4, PMID: 10704786

[10] Cardoso HD Cabral EV Vieira-Filho LD Vieyra A Paixao AD. Fetal development and renal function in adult rats prenatally subjected to sodium overload. Pediatr Nephrol. (2009) 24:1959–65. doi: 10.1007/s00467-009-1247-1, PMID: 19603192

[11] Rocco L Gil FZ da Fonseca Pletiskaitz TM de Fatima Cavanal M Gomes GN. Effect of sodium overload on renal function of offspring from diabetic mothers. Pediatr Nephrol. (2008) 23:2053–60. doi: 10.1007/s00467-008-0884-0, PMID: 18574600

[12] Levi AJ Dalton GR Hancox JC Mitcheson JS Issberner J Bates JA. Role of intracellular sodium overload in the genesis of cardiac arrhythmias. J Cardiovasc Electrophysiol. (1997) 8:700–21. doi: 10.1111/j.1540-8167.1997.tb01834.x, PMID: 9209972

[13] Roson MI Della Penna SL Cao G Gorzalczany S Pandolfo M Toblli JE. Different protective actions of losartan and tempol on the renal inflammatory response to acute sodium overload. J Cell Physiol. (2010) 224:41–8. doi: 10.1002/jcp.22087, PMID: 20232302

[14] Schorn C Frey B Lauber K Janko C Strysio M Keppeler H. Sodium overload and water influx activate the nalp3 inflammasome. J Biol Chem. (2011) 286:35–41. doi: 10.1074/jbc.M110.139048, PMID: 21051542 PMC3012992

[15] Michaels AM Zoccarato A Hoare Z Firth G Chung YJ Kuchel PW. Disrupting na(+) ion homeostasis and na(+)/K(+) atpase activity in breast cancer cells directly modulates glycolysis *in vitro* and *in vivo* . Cancer Metab. (2024) 12:15. doi: 10.1186/s40170-024-00343-5, PMID: 38783368 PMC11119389

[16] Wang Q Zheng C Hou H Bao X Tai H Huang X. Interplay of sphingolipid metabolism in predicting prognosis of gbm patients: towards precision immunotherapy. J Cancer. (2024) 15:275–92. doi: 10.7150/jca.89338, PMID: 38164288 PMC10751665

[17] Liberzon A Birger C Thorvaldsdottir H Ghandi M Mesirov JP Tamayo P. The molecular signatures database (Msigdb) hallmark gene set collection. Cell Syst. (2015) 1:417–25. doi: 10.1016/j.cels.2015.12.004, PMID: 26771021 PMC4707969

[18] Hanzelmann S Castelo R Guinney J. Gsva: gene set variation analysis for microarray and rna-seq data. BMC Bioinf. (2013) 14:7. doi: 10.1186/1471-2105-14-7, PMID: 23323831 PMC3618321

[19] Ponce-Bobadilla AV Schmitt V Maier CS Mensing S Stodtmann S. Practical guide to shap analysis: explaining supervised machine learning model predictions in drug development. Clin Transl Sci. (2024) 17:e70056. doi: 10.1111/cts.70056, PMID: 39463176 PMC11513550

[20] Chintalaramulu N Singh DP Sapkota B Raman D Alahari S Francis J. Caveolin-1: an ambiguous entity in breast cancer. Mol Cancer. (2025) 24:109. doi: 10.1186/s12943-025-02297-8, PMID: 40197489 PMC11974173

[21] Huang T Tworoger SS Hecht JL Rice MS Sood AK Kubzansky LD. Association of ovarian tumor beta2-adrenergic receptor status with ovarian cancer risk factors and survival. Cancer Epidemiol Biomarkers Prev. (2016) 25:1587–94. doi: 10.1158/1055-9965.EPI-16-0534, PMID: 27587791 PMC5135562

[22] Garlapati C Joshi S Bhattarai S Krishnamurthy J Turaga RC Nguyen T. Plk1 and aurkb phosphorylate survivin differentially to affect proliferation in racially distinct triple-negative breast cancer. Cell Death Dis. (2023) 14:12. doi: 10.1038/s41419-022-05539-5, PMID: 36627281 PMC9832024

[23] Puppe J Opdam M Schouten PC Jozwiak K Lips E Severson T. Ezh2 is overexpressed in brca1-like breast tumors and predictive for sensitivity to high-dose platinum-based chemotherapy. Clin Cancer Res. (2019) 25:4351–62. doi: 10.1158/1078-0432.CCR-18-4024, PMID: 31036541

[24] Glaviano A Foo ASC Lam HY Yap KCH Jacot W Jones RH. Pi3k/akt/mtor signaling transduction pathway and targeted therapies in cancer. Mol Cancer. (2023) 22:138. doi: 10.1186/s12943-023-01827-6, PMID: 37596643 PMC10436543

[25] Paraiso KH van der Kooi K Messina JL Smalley KS. Measurement of constitutive mapk and pi3k/akt signaling activity in human cancer cell lines. Methods Enzymol. (2010) 484:549–67. doi: 10.1016/B978-0-12-381298-8.00027-7, PMID: 21036250 PMC4001792

[26] Jeffs GJ Meloni BP Bakker AJ Knuckey NW. The role of the na(+)/ca(2+) exchanger (Ncx) in neurons following ischaemia. J Clin Neurosci. (2007) 14:507–14. doi: 10.1016/j.jocn.2006.07.013, PMID: 17430774

[27] Ho SY Phua K Wong L Bin Goh WW. Extensions of the external validation for checking learned model interpretability and generalizability. Patterns (N Y). (2020) 1:100129. doi: 10.1016/j.patter.2020.100129, PMID: 33294870 PMC7691387

[28] Fragomeni SM Sciallis A Jeruss JS. Molecular subtypes and local-regional control of breast cancer. Surg Oncol Clin N Am. (2018) 27:95–120. doi: 10.1016/j.soc.2017.08.005, PMID: 29132568 PMC5715810

[29] Lin A Zhang X Ruan YY Wang Q Zhou WJ Yan WH. Hla-F expression is a prognostic factor in patients with non-small-cell lung cancer. Lung Cancer. (2011) 74:504–9. doi: 10.1016/j.lungcan.2011.04.006, PMID: 21561677

[30] Lin A Yan WH. The emerging roles of human leukocyte antigen-F in immune modulation and viral infection. Front Immunol. (2019) 10:964. doi: 10.3389/fimmu.2019.00964, PMID: 31134067 PMC6524545

[31] Zhao L Lei W Deng C Wu Z Sun M Jin Z. The roles of liver X receptor alpha in inflammation and inflammation-associated diseases. J Cell Physiol. (2021) 236:4807–28. doi: 10.1002/jcp.30204, PMID: 33305467

[32] Wang B Tontonoz P. Liver X receptors in lipid signalling and membrane homeostasis. Nat Rev Endocrinol. (2018) 14:452–63. doi: 10.1038/s41574-018-0037-x, PMID: 29904174 PMC6433546

[33] Gong H Guo P Zhai Y Zhou J Uppal H Jarzynka MJ. Estrogen deprivation and inhibition of breast cancer growth *in vivo* through activation of the orphan nuclear receptor liver X receptor. Mol Endocrinol. (2007) 21:1781–90. doi: 10.1210/me.2007-0187, PMID: 17536009

[34] Vedin LL Lewandowski SA Parini P Gustafsson JA Steffensen KR. The oxysterol receptor lxr inhibits proliferation of human breast cancer cells. Carcinogenesis. (2009) 30:575–9. doi: 10.1093/carcin/bgp029, PMID: 19168586

[35] Nguyen-Vu T Vedin LL Liu K Jonsson P Lin JZ Candelaria NR. Liver X receptor ligands disrupt breast cancer cell proliferation through an E2f-mediated mechanism. Breast Cancer Res. (2013) 15:R51. doi: 10.1186/bcr3443, PMID: 23809258 PMC4053202

[36] Peng X Shi J Zhao Z Tong R Zhang X Zhong L. Emetine, a small molecule natural product, displays potent anti-gastric cancer activity via regulation of multiple signaling pathways. Cancer Chemother Pharmacol. (2023) 91:303–15. doi: 10.1007/s00280-023-04521-y, PMID: 36941385 PMC10027284

[37] Silva LC Borgato GB Wagner VP Martins MD Rocha GZ Lopes MA. Cephaeline is an inductor of histone H3 acetylation and inhibitor of mucoepidermoid carcinoma cancer stem cells. J Oral Pathol Med. (2022) 51:553–62. doi: 10.1111/jop.13252, PMID: 34661317 PMC9013730

